# TEMPROT: protein function annotation using transformers embeddings and homology search

**DOI:** 10.1186/s12859-023-05375-0

**Published:** 2023-06-08

**Authors:** Gabriel B. Oliveira, Helio Pedrini, Zanoni Dias

**Affiliations:** Institute of Computing, University of Campinas, Campinas, Brazil

**Keywords:** Protein function prediction, Natural language processing, Transformers

## Abstract

**Background:**

Although the development of sequencing technologies has provided a large number of protein sequences, the analysis of functions that each one plays is still difficult due to the efforts of laboratorial methods, making necessary the usage of computational methods to decrease this gap. As the main source of information available about proteins is their sequences, approaches that can use this information, such as classification based on the patterns of the amino acids and the inference based on sequence similarity using alignment tools, are able to predict a large collection of proteins. The methods available in the literature that use this type of feature can achieve good results, however, they present restrictions of protein length as input to their models. In this work, we present a new method, called TEMPROT, based on the fine-tuning and extraction of embeddings from an available architecture pre-trained on protein sequences. We also describe TEMPROT+, an ensemble between TEMPROT and BLASTp, a local alignment tool that analyzes sequence similarity, which improves the results of our former approach.

**Results:**

The evaluation of our proposed classifiers with the literature approaches has been conducted on our dataset, which was derived from CAFA3 challenge database. Both TEMPROT and TEMPROT+ achieved competitive results on $F_{\max }$, $S_{\min }$, AuPRC and IAuPRC metrics on Biological Process (BP), Cellular Component (CC) and Molecular Function (MF) ontologies compared to state-of-the-art models, with the main results equal to 0.581, 0.692 and 0.662 of $F_{\max }$ on BP, CC and MF, respectively.

**Conclusions:**

The comparison with the literature showed that our model presented competitive results compared the state-of-the-art approaches considering the amino acid sequence pattern recognition and homology analysis. Our model also presented improvements related to the input size that the model can use to train compared to the literature methods.

**Supplementary Information:**

The online version contains supplementary material available at 10.1186/s12859-023-05375-0.

## Background

With the development of sequencing technologies in the last decades, a large number of proteins have been sequenced. On the other hand, the analysis of the specific characteristics of each one is still far from the number of sequenced proteins, mainly due to the effort of time and money required by laboratorial experiments compared to sequencing techniques. Due to this fact, works in the literature have been proposing computational methods to predict this type of information from sequenced proteins, such as secondary structures [1] and functions [2], in order to decrease this gap [3].

The protein function annotation task uses Gene Ontology (GO) [4] to evaluate the predictions made in three different ontologies, Biological Process (BP), which represents the process that proteins are involved, Cellular Component (CC), which is the place in the cell where the protein performs the function, and Molecular Function (MF), the function played by the protein at a molecular level. In all of them, each protein can have a different assigned function, which makes this task a multi-label prediction. Furthermore, the organization of the ontologies is in a direct acyclic graph, with the deeper terms being more specific than the shallow ones and, if a protein has a specific term, it also has all the ancestor ontology terms up to the root node.

In the literature, different approaches considering a huge type of features have been presented for the protein function annotation task, such as amino acid sequence pattern recognition [2, 5–7], sequence similarity analysis using homology search [8, 9] based on BLASTp [10] or DIAMOND [11], which are local alignment tools, structure [12, 13], protein-protein network interaction [13, 14], biological features [15, 16], text mining from scientific articles [17], and combination of them [18, 19]. Compared to the other features, protein sequence is the most common information available about proteins, so methods that use it, such as amino acid sequence pattern analysis and homology search, can predict a large collection of proteins compared to models that apply other input characteristics to their models.

In this paper, we present two protein function annotation models, based on protein pattern analysis and homology search. The first one is TEMPROT, a method that uses the amino acid sequence to make GO predictions based on the fine-tuning and extraction of embeddings from ProtBERT-BFD [20], a Transformer [21] architecture pre-trained in protein sequences. As an evolution of TEMPROT, we developed TEMPROT+, an ensemble of the former approach with BLASTp, responsible for making homology search based on local sequence similarity.

During the evaluation on our dataset, which is based on CAFA3 challenge, we compared TEMPROT and TEMPROT+ against state-of-the-art approaches using amino acid sequence pattern recognition and homology search. We applied DeepGO [5], DeepGOPlus [6], TALE+ [2], ATGO+ [7], and the baseline models proposed in the CAFA challenge on our data. Our methods achieved the best $F_{\max }$ on CC and MF ontologies, and competitive results on AuPRC, IAuPRC, and $S_{\min }$ metrics on the test set, able to predict rare terms in all three ontologies and competitive results considering the Eukaryota, Bacteria, and Archaea domains.

Our main contributions are: (1) we report issues on the main dataset available in the literature for protein function annotation and we create a new version of this dataset, without these issues, (2) we propose a new metric that showed to be fairer in the evaluation of precision and recall curves, (3) we present a new method to generate artificial proteins for training data enhancement based on PAM matrix [22], improving the results compared to the standard version, that is, without this technique and (4) unlike state-of-the-art methods, our method can use sequences without length restriction.

## Methods

In this section, we present the dataset applied in our experiments, describe our model and the comparison methods, and detail the evaluation metrics.

### Dataset

The dataset employed to evaluate our model and compare with the literature was generated by DeepGOPlus [6] work based on CAFA3 [23], which is the most recent dataset from CAFA challenge that has a published paper reporting the official methods and results. The split of the database considered the timestamp, that is, the training and validation sets have proteins with experimental annotations published before September 2016, and the test set contains proteins with experimental annotations published between September 2016 and November 2017.

During the exploration of the dataset, we noticed that some sequences are identical on different sets considering the same ontology, even with different functions annotated. Therefore, to deal with this issue related to data leakage about protein sequence, we removed the duplicated data in the following steps: (1) exclusion of duplicated sequences from the training set, considering the test set, (2) removal of duplicated sequences from the validation set, considering the test set, (3) exclusion of duplicated sequences from the validation set, considering the training set. Considering the duplicated sequences in the training and test set, we removed 430 (0.89%), 164 (0.36%), and 47 (0.14%) sequences out of the 48,121, 45,473 and 32468 sequences from the original training set of BP, CC and MF, respectively, with 60.9% (BP), 87.8% (CC) and 74.5% (MF) of these removed sequences having different labels in these two sets.

As a final preprocessing step of the dataset, we considered only terms presented in at least 50 proteins as possible labels in the annotation task, as used in the DeepGOPlus work. The

**Table 1:** Number of proteins and functions in BP, CC and MF ontologies in the dataset

	BP	CC	MF
Training set	47,691	45,309	32,421
Validation set	5252	4985	3587
Test set	2392	1265	1137
Functions	3992	551	677

number of proteins in each set and the number of functions in each ontology are presented in Table 1.

### TEMPROT

**Fig. 1:**
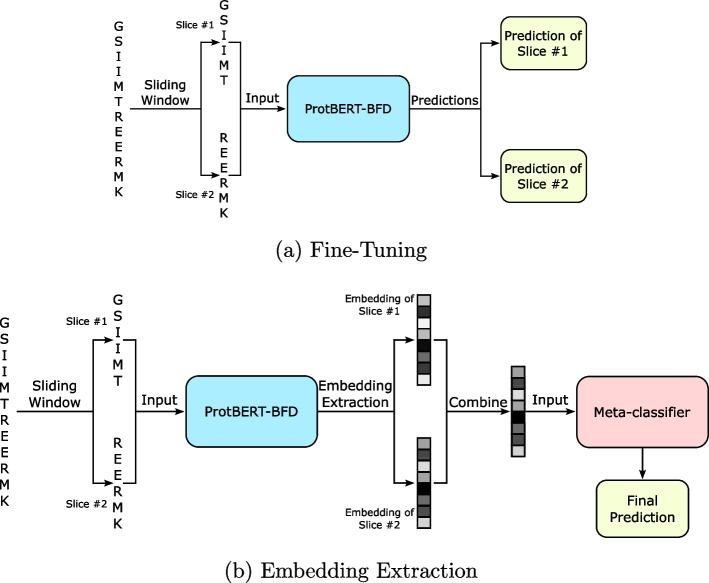
The pipeline of the Transformer-based EMbeddings for PROTein function annotation (TEMPROT). **a** Each sequence is split into slices using the sliding window technique and fined-tune ProtBERT-BFD backbone. **b** With the fine-tuned ProtBERT-BFD, all the slices pass through the backbone to extract the embeddings from the last representation of CLS token, then combine the embeddings to have a unique representation of the protein and make the final prediction with the meta-classifier

In this subsection, we describe our protein sequence-based method for annotating protein functions, which we called Transformer-based EMbeddings for PROTein function annotation (TEMPROT). Figure 1 illustrates TEMPROT pipeline.

#### Fine-tuning

Following state-of-the-art natural language processing techniques, we fine-tuned ProtBERT-BFD [20], a BERT-based [24] model pre-trained on BFD dataset [25], and used it as extractor of features from the protein sequence for function annotation.

**Fig. 2:**
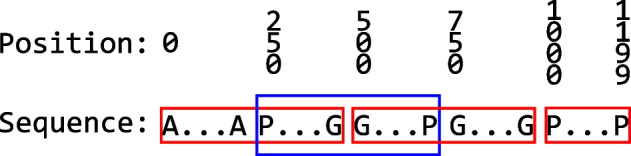
Example of sliding window technique using a protein with 1200 amino acids. The red squares represent the standard slices, and the blue square illustrates the additional slice

As ProtBERT-BFD is a BERT-based architecture, it cannot cope with sequences longer than 512 amino acid tokens during fine-tuning for sequence classification task, due to the quadratic memory limitations of the attention mechanisms, requiring large computational resources for longer inputs. Based on this fact, we split the protein sequences using a sliding window technique of size of 500 amino acids without superposition. To improve the generalization of the model, we proposed additional slices in the case of two consecutive slices have at least 250 amino acids. In this case, we created an additional slice with the 250 last amino acids of the first slice and the 250 first amino acids of the last slice. During this process, we assigned the same labels from the original protein sequence to all the slices generated. Figure 2 shows an example of this approach with a protein with 1200 amino acids, with the red squares representing the standard slices and the blue square as the additional slice. In the example, the first and the second standard slices have the size equal to 500, and the last has the size equal of 200, and based on this, it is possible to create just one additional slice.

In TEMPROT, the first step is the fine-tuning process of ProtBERT-BFD in the protein function annotation task, as described in part (a) of Fig. 1. To do so, we passed each input data, that is, the slices that were generated using the sliding window technique, through the backbone. It is important to notice that it is possible to make predictions using the backbone (part (a) of Fig. 1). We compare and discuss the results based on the predictions made in this first step with the final model in the Results section.

During the fine-tuning step, we used ProtBERT-BFD model available at Hugging Face [26] repository, with TensorFlow [27] and ktrain [28] libraries. We fine-tuned the model during 10 epochs with the early stopping technique, binary cross-entropy loss function, and Adam [29] optimizer.

#### Embedding extraction

After the fine-tuning process, we used the backbone architecture as feature extraction. To do so, we passed all sequence slices through the fine-tuned ProtBERT-BFD backbone and extracted the embeddings from CLS token from the last encoder block of the fine-tuned architecture. This token is responsible for gathering the context of the sentence, that is, it is used as a special token for classification tasks. Based on that, we extracted the embeddings from the deepest representation of this token. As a result, each slice generated a feature vector of 1024 float values.

Then, we aggregated the embeddings from the slice of the same protein to have a unique feature vector of size 1024 for each protein. To do so, we applied the mean operation between the embeddings of all the slices of the same protein.

#### Meta-classifier

As the last step of our method, we employed each protein representation in a meta-classifier, which is responsible for making the final prediction.

For the meta-classifier, we constructed a multi-layer perceptron neural network model with TensorFlow library. The architecture consisted of one hidden layer with 1000 neurons and ReLU activation. We trained the model during 100 epochs with early stopping and reduction of learning rate on plateau techniques, binary cross-entropy loss function, and Adam optimizer.

#### Data augmentation

During all the steps of TEMPROT, we employed data augmentation for the training set. Inspired by EDA technique [30], for each protein in the training set, we created a copy of it and made substitutions of amino acids considering the PAM1 matrix [22] in an offline manner.

In the PAM matrix *M*, each row *i* and column *j* represents the amino acids, where a specific position $M_{ij}$ indicates the likelihood of substitution of amino acid *j* per amino acid *i*. It is important to note that, in the PAM matrix, the most likely substitution of a specific amino acid is for the same amino acid, that is, the substitution does not change the amino acid.

For the substitutions, we considered Eq. 1, where the number of substitutions of a protein *p* is equal to its length *L* and a constant *k*. We explored different values for *k*, and the best results were achieved with *k* equal to 2. With this set up, the augmented data changed $2.03\% \pm 0.84\%$, $2.04\% \pm 0.84\%$, and $2.03\% \pm 0.78\%$ from the original training data for BP, CC and MF ontologies, respectively. We investigate the impact of the usage of data augmentation in the Results section.

1 $$\begin{aligned} {\text {Subs}}(p) = L \times k \end{aligned}$$

### TEMPROT+

In this subsection, we present TEMPROT+, an ensemble of TEMPROT with BLASTp [10], a homology search tool.

#### BLASTp

Considering the improvements obtained by TALE+ and DeepGOPlus using DIAMOND, and by ATGO+ using BLASTp, we also implemented a version of our method combined with a homology search using BLASTp. To do so, we ran BLASTp to perform homology search considering sequence similarity of validation and test proteins against the sequences from the training set. As used in the previous methods, we set the *E*-value parameter equal to 0.001.

Based on the retrieved sequences, we applied the bitscore to make the predictions, as presented in Eq. 2, where *S*(*p*, *f*) indicates a score prediction for a protein *p* and a specific function *f*, *s* is a protein of the set *E* of retrieved proteins of the training set, $T_s$ is the functions played by *s*, and *I*() is a function that returns 1 if the condition inside is true or 0 if it is false.

2 $$\begin{aligned} S(p, f) = \frac{\sum _{s \in E} I(f \in T_s) \times {\text {bitscore}}(p, s)}{\sum _{s \in E} {\text {bitscore}}(p, s)} \end{aligned}$$

#### Ensemble of TEMPROT and BLASTp

To ensemble TEMPROT and BLASTp predictions, we investigated various linear combination approaches between their predictions, as shown in Additional file 1. The ensemble method applied in our model is expressed in Eq. 3, which achieved the best results compared to variations of this equation, where *S*(*p*, *f*) indicates a score prediction for a protein *p* and a specific function *f*, considering the prediction $y_{T}$ from TEMPROT and $y_{B}$ from BLASTp.

3 $$\begin{aligned} S(p,f) = \alpha \times y_{T} + (1 - \alpha ) \times y_{B} \end{aligned}$$

In order to find the $\alpha$ values for each ontology, we ran a grid search considering the validation set. The best outcomes were obtained for $\alpha$ equal to 0.21, 0.60, and 0.30 for BP, CC, and MF, respectively.

### Comparison methods

In order to compare our method with state-of-the-art models, we selected three different types of approaches. We describe each one as follows.

The first approach is the baseline methods, as proposed by the CAFA challenge organizers [23]. There are two classifiers in this category, naive and based on sequence similarity using BLAST. Naive one is a classifier that predicts that each function of proteins in the test set has the same chance, that is, the same relative frequency, of the same function in the training set. For the second baseline classifier, we ran BLASTp for the prediction based on sequence similarity analysis using the highest local alignment sequence hit. In the Results section, we call the BLASTp implementation of CAFA as CAFA-BLASTp.

To evaluate TEMPROT considering the state-of-the-art approaches, we also assessed methods that employ amino acid sequence pattern recognition to make predictions. With that, we compared our outcomes with DeepGO, DeepGOPlusCNN, TALE+Transformers and ATGO.

DeepGO [5] is a method that applies protein sequence and protein network features to convolutional neural networks. To make a fair comparison, we employed only the protein sequence part in the evaluation. DeepGOPlusCNN [6] is an evolution of DeepGO, capable of outperforming the previous method with architecture and preprocessing steps. We also compared our results with Transformer-based methods, that is, TALE+Transformers [2], an approach based on the ensemble of different configurations of the original Transformer architecture, and ATGO [7], a method that extracts embeddings from ESM-1b [31] architecture.

The last models we compared are based on the ensemble of sequence pattern recognition and homology search predictions. We assessed DeepGOPlus [6], an ensemble of DeepGOPlusCNN with DIAMOND, TALE+ [2], an ensemble of TALE+Transformers and DIAMOND and ATGO+ [7], an ensemble of ATGO and BLASTp, with TEMPROT+. We also evaluated DIAMOND and BLASTp predictions based on Eq. 2.

For all approaches, we followed the hyperparameters reported in their original papers and the code available in their respective repositories and ran each one in our dataset in order to have a fair comparison with our results.

### Evaluation

To evaluate and compare our model with the literature, we used four evaluation metrics. The first one is $F_{\max }$, the official metric of CAFA challenge [23]. $F_{\max }$ measures the maximum harmonic mean between precision and recall considering the predictions in all thresholds $\tau$ from 0 up to 1 with steps of 0.01. Equations 4, 5, and 6 represents precision at $\tau$, recall at $\tau$ and $F_{\max }$, respectively, where *f* is a function of the ontology that is in evaluation, $P_i(\tau )$ is the set of functions predicted in the threshold $\tau$ for a protein *i*, $T_i$ is the ground truth of a protein *i*, $m(\tau )$ is the number of proteins with at least one prediction equal to or greater than the threshold $\tau$, $n_e$ is the number of proteins considering during the evaluation, and *I*() is a function that returns 1 if the condition inside is true or 0 if it is false.

4 $$\begin{aligned} {\text {pr}}(\tau )= & {} \frac{1}{m(\tau )} \sum ^{m(\tau )}_{i=1} \frac{\sum _{f} I (f \in P_i(\tau ) \wedge f \in T_i)}{\sum _{f} I (f \in P_i(\tau ))} \end{aligned}$$

5 $$\begin{aligned} {\text {rc}}(\tau )= & {} \frac{1}{n_e} \sum ^{n}_{i=1} \frac{\sum _{f} I (f \in P_i(\tau ) \wedge f \in T_i)}{\sum _{f} I (f \in T_i)} \end{aligned}$$

6 $$\begin{aligned} F_{\max }= & {} \max _{\tau } \left\{ \frac{2 \times {\text {pr}}(\tau ) \times {\text {rc}}(\tau )}{{\text {pr}}(\tau ) + {\text {rc}}(\tau )} \right\} \end{aligned}$$

Based on the precision and recall values calculated in $F_{\max }$, we assessed the area under the precision–recall curve (AuPRC) of the methods. This metric is common in the literature to evaluate the protein function prediction task.

The main problem of AuPRC is that it penalizes when a method can only make predictions with high recall and high precision compared to methods that can predict with a long range of precision and recall values. Therefore, we propose a new evaluation metric, the interpolated area under the precision–recall curve (IAuPRC). IAuPRC applies the interpolation for AuPRC, making evaluation more reliable and without penalization for methods that can predict functions with always good precision and recall values. The interpolation is represented by Eq. 7, where the precision value at a specific recall *P*(*R*) is equal to the maximum value of precisions with greater or equal recall levels $P(R')$, where $R' \geqslant R$.

7 $$\begin{aligned} P(R) = \max P(R') \end{aligned}$$

**Fig. 3:**
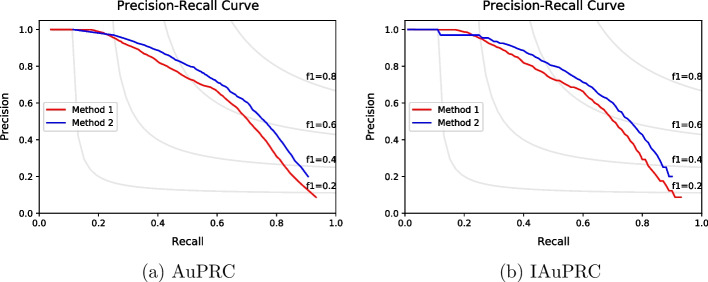
Differences for AuPRC and IAuPRC metrics

Figure 3 shows an example of two methods considering AuPRC and IAuPRC. In AuPRC analysis, method 1 achieved 0.618 in this metric, while method 2 obtained 0.584. However, it is clear that the method 2’s curve is better than method 1’s curve, and method 2 has been penalized by not making predictions with lower (worst) recall values. In the IAuPRC analysis, the interpolation of both curves resulted in 0.643 of IAuPRC for method 1 and 0.683 of IAuPRC for method 2, which indicates that method 2 is superior than method 1.

The last metric is $S_{\min }$, which measures the semantic distance considering the information content (${\text {IC}}$) of each function that the prediction of false positive (${\text {mi}}$) and false negative (${\text {ru}}$) in a specific $\tau$, where *Pb*(*f*|*Pr*(*f*)) is the probability of a function *f* given set of parents *Pr*(*f*). Equations 8, 9, 10, and 11 describe ${\text {IC}}$, ${\text {ru}}$, ${\text {mi}}$, and $S_{\min }$, respectively.

8 $$\begin{aligned} {\text {IC}}(f)= & {} -\log (Pb(f|Pr(f)) \end{aligned}$$

9 $$\begin{aligned} {\text {ru}}(\tau )= & {} \frac{1}{n_e} \sum ^{n_e}_{i=1} \sum _{f \in T_i - P_i(\tau )} {\text {IC}}(f) \end{aligned}$$

10 $$\begin{aligned} {\text {mi}}(\tau )= & {} \frac{1}{n_e} \sum ^{n_e}_{i=1} \sum _{f \in P_i(\tau ) - T_i} {\text {IC}}(f) \end{aligned}$$

11 $$\begin{aligned} S_{\min }= & {} \min _{\tau } \sqrt{{\text {ru}}(\tau )^2 + {\text {mi}}(\tau )^2} \end{aligned}$$

It is important to note that these metrics comply with the ontology format, that is, if a given term is predicted by a classifier in a specific threshold $\tau$, all the ancestors are also predicted in this threshold.

## Results

In this section, we present and discuss the results of TEMPROT and TEMPROT+ compared to the literature.

### Evaluation on test set

We evaluated TEMPROT and TEMPROT+ and compared the results with the state-of-the-art methods, considering the test set of our dataset. Table 2 presents the outcomes of each model.

**Table 2:** Evaluation of TEMPROT and TEMPROT+ compared to the state-of-the-art approaches on the test set using $F_{\max }$, AuPRC, IAuPRC and $S_{\min }$ evaluation metrics

Method	*F*_max_			AuPRC			IAuPRC			*S*_min_		
	BP	CC	MF	BP	CC	MF	BP	CC	MF	BP	CC	MF
Naive	0.402	0.611	0.446	0.266	0.521	0.228	0.345	0.634	0.370	25.423	10.268	9.349
CAFA-BLASTp	0.468	0.469	0.551	0.208	0.215	0.287	0.215	0.216	0.296	38.083	18.755	9.124
DeepGO	0.337	0.379	0.489	0.247	0.257	0.309	0.304	0.382	0.465	27.414	11.880	8.821
DeepGOPlusCNN	0.498	0.664	0.531	0.444	0.637	0.460	0.465	0.634	0.528	23.799	9.783	8.240
TALE+Transformers	0.491	0.661	0.550	0.477	0.613	0.444	0.469	0.706	0.549	23.929	9.682	8.115
ATGO	**0.547**	0.684	0.616	**0.506**	**0.667**	**0.623**	**0.524**	**0.724**	0.632	**22.228**	9.437	7.228
TEMPROT	0.499	**0.689**	**0.643**	0.459	0.639	0.561	0.483	0.719	**0.664**	23.652	**9.209**	**6.973**
DIAMOND	0.519	0.593	0.572	0.286	0.237	0.320	0.417	0.483	0.462	23.066	9.957	7.164
BLASTp	0.561	0.637	0.620	0.402	0.380	0.360	0.502	0.586	0.562	22.183	9.795	6.805
DeepGOPlus	0.553	0.677	0.619	0.514	0.638	0.559	0.536	0.717	0.635	22.648	9.515	7.090
TALE+	0.555	0.681	0.631	0.547	0.643	0.621	0.540	0.724	0.643	22.615	9.363	6.949
ATGO+	**0.589**	0.690	0.652	**0.550**	**0.660**	**0.650**	**0.571**	**0.731**	**0.689**	**21.233**	9.286	**6.617**
TEMPROT+	0.581	**0.692**	**0.662**	0.529	0.641	0.595	0.558	0.728	**0.689**	21.892	**9.169**	6.662

The best results of each metric for sequence pattern recognition and ensemble of sequence pattern recognition and homology search analysis are highlighted

TEMPROT achieved the best $F_{\max }$ and $S_{\min }$ compared to sequence pattern recognition approaches (DeepGO, DeepGOPlusCNN, TALE+Transformers, and ATGO) on CC and MF ontologies, outperforming ATGO by 0.005 on CC and 0.027 on MF of $F_{\max }$. Considering IAuPRC, TEMPROT achieved competitive results, with the best outcomes on MF ontology.

In the second analysis, with methods based on the ensemble of predictions of sequence pattern recognition and homology search, TEMPROT+ achieved the best results on $F_{\max }$ on CC and MF, with improvements of 0.002 and 0.010 on CC and MF, respectively, compared to ATGO+, the second best outcomes. Considering $S_{\min }$, TEMPROT+ obtained the best CC outcomes and the second best on BP and MF ontologies.

### Domain generalization

To analyze the predictions of each model on the test set on different domains, we assessed each approach on Eukaryota, Bacteria, and Archaea with $F_{\max }$ evaluation metric for each ontology.

**Fig. 4:**
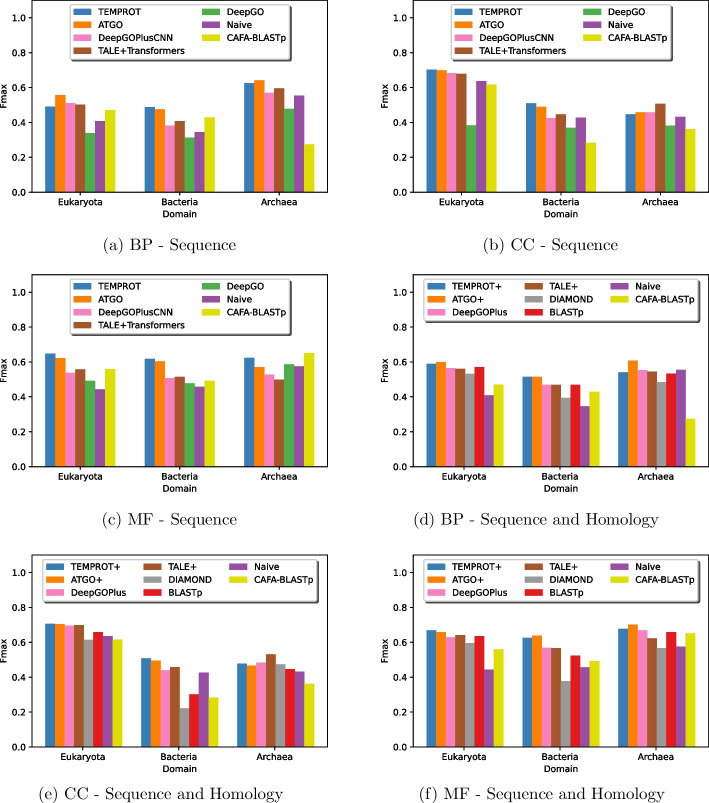
Comparions of TEMPROT and TEMPROT+ with the state-of-the-art on domain evaluation

Figure 4 illustrates the $F_{\max }$ value of each method on each domain. The outcomes show that TEMPROT had the best results on MF ontology, as well as competitive scores on BP and CC ontologies, with the best $F_{\max }$ on Bacteria domain on BP, and the best $F_{\max }$ on Eukaryota and Bacteria domains on CC. TEMPROT+ achieved the best results on Eukaryota domain on MF ontology, as well as the best results on Bacteria and competitive results on Eukaryota and Archaea domains on BP and CC ontologies.

### Frequency analysis

**Fig. 5:**
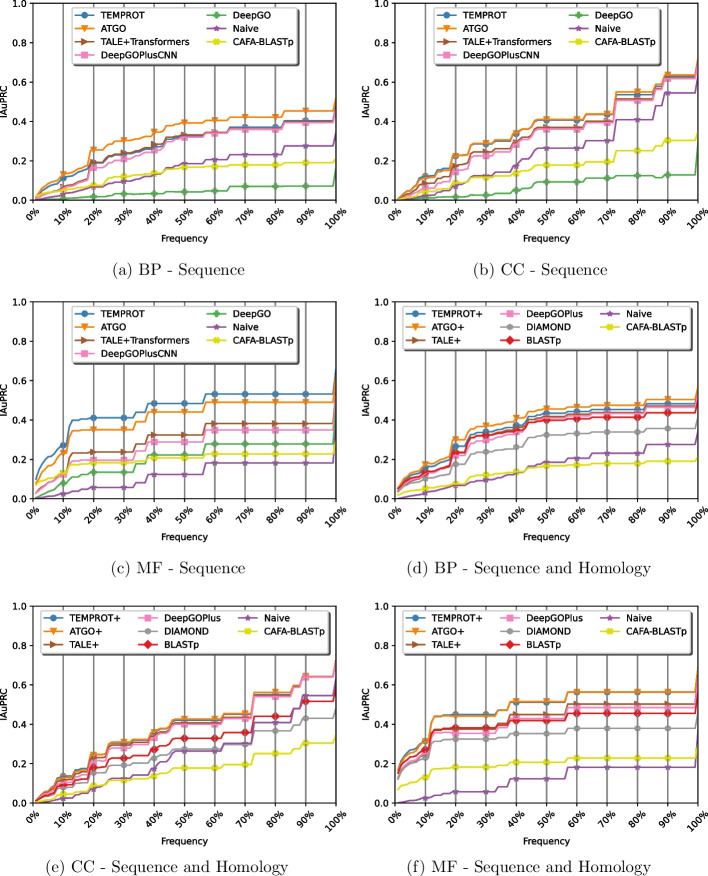
Comparions of TEMPROT and TEMPROT+ with the state-of-the-art on frequency analysis

An important aspect is that models must correctly predict rare terms. To analyze the ability of the approaches to annotate terms with different frequency appearances, we evaluated each model by considering IAuPRC on all 100 percentage values on the proteins of the test set, as shown in Fig. 5. In the evaluation, if we were analyzing a specific percentage, all terms that have up to that frequency are analyzed, for instance, for a 10% analysis, ontologies terms that have up to 10% of frequency are considered.

As a result, TEMPROT achieved the best overall results on MF ontology, with the highest values in all frequencies, and competitive results with ATGO on CC ontology. In the case of the BP ontology, TEMPROT had the second best performance at the beginning of the analysis, that is, in rare functions, with competitive results for the other values.

Considering TEMPROT+, across all ontologies, our method had the best performance in rare terms, with competitive outcomes for other values. Concerning MF ontology, TEMPROT+ obtained the best overall outcomes along with ATGO+.

### Ablation study

To evaluate the impact of doing the fine-tuning of ProtBERT-BFD, applying data augmentation to the sequences, and using the meta-classifier, we assessed different configurations, as shown in Table 3, on the test set.

**Table 3:** Ablation study of different configurations of our method on the three ontologies

Method	$F_{\max }$		
	BP	CC	MF
TEMPROT+	0.581	0.692	0.662
TEMPROT	0.499	0.689	0.643
TEMPROT w/o augmentation	0.493	0.687	0.639
TEMPROT w/o fine-tuning	0.493	0.681	0.618
TEMPROT w/o augmentation and w/o fine-tuning	0.490	0.681	0.620
TEMPROT w/o meta-classifier	0.477	0.677	0.592

The ablation study indicates that the use of the meta-classifier (second row of Table 3) in our method achieved better results compared to the prediction based on the backbone only (part (a) of Fig. 1, indicated in the last row of Table 3). It is important to note that we needed to aggregate the predictions of slices from the same protein to have a unique prediction per protein at without meta-classifier approach. To do so, we applied the mean operation on the predictions considering all the slices of the same protein.

Concerning data augmentation and fine-tuning, the version without data augmentation and without fine-tuning had the worst performance based on $F_{\max }$ (TEMPROT without augmentation and without fine-tuning). The application of fine-tuning technique (TEMPROT without augmentation) or data augmentation (TEMPROT without fine-tuning) improved the results compared to the former configuration in general. TEMPROT, a version using both fine-tuning and data augmentation, achieved the best result considering the sequence pattern information, which represents that both techniques are important for the outcomes. In the end, the best $F_{\max }$ was achieved by TEMPROT+, showing that the ensemble of machine learning with homology search predictions can indeed improve the results.

### Prediction time

We assessed TEMPROT and TEMPROT+ compared to the literature considering the average time to predict each protein of the test set of each ontology. Table 4 presents the results, showing that convolutional-based methods, such as DeepGOPlusCNN and DeepGO, are more efficient than Transformer-based models, such as ATGO and TEMPROT. Concerning homology-based predictions by different tools, DIAMOND required less time to execute than BLASTp, which impacted the runtime of DeepGOPlus and TALE+ compared to TEMPROT+ and ATGO+.

**Table 4:** Average prediction time in seconds for each protein of the test set on BP, CC and MF ontologies

Method	BP	CC	MF
Naive	0.001	0.002	0.002
DIAMOND	0.005	0.008	0.007
DeepGOPlusCNN	0.013	0.022	0.025
DeepGO	0.019	0.025	0.026
DeepGOPlus	0.023	0.030	0.032
TALE+Transformers	0.035	0.047	0.062
TALE+	0.040	0.055	0.069
ATGO	0.304	0.508	0.434
TEMPROT	0.617	0.643	0.627
BLASTp	0.666	0.946	0.595
CAFA-BLASTp	0.666	0.946	0.595
ATGO+	0.971	1.455	1.029
TEMPROT+	1.283	1.589	1.222

## Discussion

The outcomes of both classifiers presented in this work surpassed the methods in the literature considering sequence pattern recognition and the ensemble of sequence information with homology search via sequence similarity. Compared to state-of-the-art methods, TEMPROT and TEMPROT+ can train using sequences of different lengths, which is not possible in the literature approaches evaluated in this paper. DeepGO and DeepGOPlus (also DeepGOPlusCNN) trained with sequences up to 1000 and 2000, respectively. In the case of TALE+ (also TALE+Transformers) and ATGO (also ATGO+), sequences longer than 1000 (TALE+) and 1022 (ATGO+) are cut into a subsequence equal to the method maximum input size.

Considering the evaluation, our methods also presented competitive results in domain generalization, with the best outcomes on MF ontology. We conclude that methods based on pre-trained on a large volume of protein sequences, that is, TEMPROT and ATGO, are able to classify protein functions better than other models, due to this ontology is more dependent of protein sequences [32]. On BP and CC ontologies, both TEMPROT and TEMPROT+ achieved the best results on at least one domain.

In the frequency analysis, TEMPROT and TEMPROT+ obtained the best results on MF and both of them achieved the best outcomes on rare terms (lower frequencies) along with ATGO on CC, and competitive results on BP. With that, the experiments indicated that methods with pre-trained architectures, such as TEMPROT and ATGO, are able to predict terms with lower frequencies.

Regarding the BP ontology, ATGO and ATGO+ outperformed TEMPROT and TEMPROT+ in most evaluations. Since this ontology has more terms than CC and MF, we conclude that it could muddle the classification of TEMPROT compared to ATGO. Furthermore, ATGO extracts embeddings from different layers of ESM-1b, which may help the generalization of this model. With that, the ensembles of sequence pattern recognition and homology search, that is, ATGO+ and TEMPROT+, follow the pattern of ATGO and TEMPROT.

We also noticed that our method has shown improvements in generalization by making fine-tuning and applying data augmentation techniques on the Transformers backbone. We demonstrated the importance of the usage of the meta-classifier during our investigation.

## Conclusions

In this work, we presented and discussed a model based on Transformer embeddings capable of annotating protein based on its sequences. Our model can also be ensembled with homology search predictions, resulting in a classifier that reported better outcomes than the standard version.

In our experiments, we showed that TEMPROT and TEMPROT+ outperformed state-of-the-art approaches on MF and CC ontologies, considering $F_{\max }$, the main metric for protein function prediction in the literature. Our method also presented improvements related to input size compared to state-of-the-art approaches.

For future improvements of TEMPROT and TEMPROT+, we can highlight the investigation of additional features, such as protein-protein interaction networks and structure information, which can help to improve the results of proteins that have this information available. We also plan to investigate different data augmentation techniques, from adding insertions and deletions in the actual approach, to exploring protein generation models. Another possible direction is the analysis of long Transformers, which can cope with sequences longer than 512 amino acids without any preprocessing step and without large computation resources, and the utilization of different configuration of windows, such as domain-based selection. We also plan experiments considering different approaches to ensemble TEMPROT with BLASTp predictions. As a final point, we intend to evaluate our method on different databases, such as other versions of CAFA dataset.

## Supplementary Information

**Additional file 1.** Analysis of ensemble techniques of TEMPROT and BLASTp.

## Data Availability

The datasets generated and analysed during the current study are available on https://zenodo.org/record/7409660. The protein function annotation method generated and analyzed during the current study is available in the Github repository: https://github.com/gabrielbianchin/TEMPROT.

## Abbreviations

AuPRC: Area under precision–recall curve
BERT: Bidirectional encoder representations from transformers
BFD: Big fantastic database
BP: Biological process
CAFA: Critical assessment of protein function annotation
CC: Cellular component
EDA: Easy data augmentation
IAuPRC: Interpolated area under precision–recall curve
MF: Molecular function
PAM: Point accepted mutation
ReLU: Rectified linear unit
TEMPROT: Transformer-based EMbeddings for PROTein function annotation
