# Supplementary material for: TEMPROT: protein function annotation using transformers embeddings and homology search
Source: BMC Bioinformatics. 2023 Jun 8;24:242. doi: 10.1186/s12859-023-05375-0 (PMC10249241; doi:10.1186/s12859-023-05375-0)
Supplement: Supplementary file 1 — Additional file 1. Analysis of ensemble techniques of TEMPROT and BLASTp. [file 12859_2023_5375_MOESM1_ESM.zip › Additional file 1/Additional file 1.pdf]

Paper Title: TEMPROT: protein function annotation using Transformers embeddings and homology search
Authors: Gabriel Bianchin de Oliveira, Helio Pedrini and Zanoni Dias

Analysis of Ensemble of TEMPROT and BLASTp

We investigated different approaches to ensemble TEMPROT and BLASTp, considering variations of linear combination of the predictions of these two classifiers.

The first one (called M1 in the comparisons), which is the method applied on DeepGOPlus, TALE+ and ATGO+, is described in Equation 1, where $S(p, f)$ indicates a score prediction for a protein p and a specific function f, considering the prediction y_T from TEMPROT and y_B from BLASTp.

$$S(p, f) = \alpha \times y_T + (1 - \alpha) \times y_B \quad (1)$$

The second approach (called M2 in the comparisons) uses the average bitscore value $bitscore_p$, calculated for each sequence p from BLASTp retrieved similar sequences, to employ a linear combination considering if $bitscore_p$ is equal or greater than a threshold $bitscore_k$ using α', otherwise using α'', as presented in Equation 2.

$$S(p, f) = \begin{cases} \alpha' \times y_T + (1 - \alpha') \times y_B, & \text{if } bitscore_p \geq bitscore_k \\ \alpha'' \times y_T + (1 - \alpha'') \times y_B, & \text{otherwise} \end{cases} \quad (2)$$

The third approach (called M3 in the comparisons) applies the linear combination if $bitscore_p$ is equal or greater than $bitscore_k$, otherwise, the importance of BLASTp predictions reduces accordingly to low $bitscore_p$ values, as shown in Equation 3.

$$S(p, f) = \begin{cases} \alpha \times y_B + (1 - \alpha) \times y_T, & \text{if } bitscore_p \geq bitscore_k \\ (\alpha \times \frac{bitscore_p}{bitscore_k}) \times y_B + (1 - (\alpha \times \frac{bitscore_p}{bitscore_k})) \times y_B, & \text{otherwise} \end{cases} \quad (3)$$

The last approach (called M4 in the comparisons) uses genetic algorithm to search weights between 0 and 1 for each labels to make linear combinations of y_T and y_B. To do so, the population starts with 100 individuals and for each generation the best 25 individuals are selected to generate 75 new individuals based on the linear combination of the parents. Each generated individuals has 10% of chance to suffer mutations, where each value of them is multiplied from 0.5 up to 2.0 based on uniform distribution.

For M1, M2 and M3 approaches, we ran all the combinations for α (or α' and α'') ranging from 0.00 up to 1.00, with step of 0.01 on the validation set. We ran 10 generations and selected the best individual for M4. Tables 1, 2 and 3 present the best results of each individual combination for each approach considering $F_{\max}$ metric. We concluded that M1, M2, M3 and M4 had similar results and we selected M1, which is the same employed by DeepGOPlus, TALE+ and ATGO+ methods, since our ensemble method due to this approach is simpler than M2, M3 and M4.

Table 1: $F_{\max}$ on the validation set of BP ontology.

Method	$bitscore_k$	α'	α''	$F_{\max}$
TEMPROT	—	—	—	0.492
BLASTp	—	—	—	0.555
M1	—	0.21	—	0.568
M2	25	1.00	0.16	0.569
M2	50	0.40	0.16	0.569
M2	75	0.26	0.02	0.569
M2	100	0.20	0.05	0.589
M2	125	0.21	0.02	0.568
M2	150	0.21	0.02	0.569
M2	175	0.21	0.02	0.568
M2	200	0.17	0.02	0.569
M3	25	0.84	—	0.569
M3	50	0.84	—	0.569
M3	75	0.89	—	0.569
M3	100	0.98	—	0.567
M3	125	0.99	—	0.566
M3	150	1.00	—	0.564
M3	175	1.00	—	0.563
M3	200	1.00	—	0.561
M4	—	—	—	0.568

Table 2: $F_{\max}$ on the validation set of CC ontology.

Method	$bitscore_k$	α'	α'	$F_{\max}$
TEMPROT	—	—	—	0.671
BLASTp	—	—	—	0.702
M1	—	0.60	—	0.742
M2	25	1.00	0.51	0.742
M2	50	0.72	0.51	0.742
M2	75	0.53	0.51	0.742
M2	100	0.55	0.51	0.742
M2	125	0.55	0.55	0.741
M2	150	0.57	0.55	0.741
M2	175	0.52	0.59	0.742
M2	200	0.52	0.60	0.742
M3	25	0.40	—	0.742
M3	50	0.40	—	0.742
M3	75	0.48	—	0.742
M3	100	0.50	—	0.741
M3	125	0.56	—	0.741
M3	150	0.69	—	0.741
M3	175	0.69	—	0.741
M3	200	0.79	—	0.740
M4	—	—	—	0.740

Table 3: $F_{\max}$ on the validation set of MF ontology.

Method	$bitscore_k$	α'	α'	$F_{\max}$
TEMPROT	—	—	—	0.731
BLASTp	—	—	—	0.696
M1	—	0.30	—	0.724
M2	25	1.00	0.28	0.724
M2	50	0.53	0.28	0.724
M2	75	0.34	0.28	0.724
M2	100	0.35	0.16	0.725
M2	125	0.26	0.28	0.724
M2	150	0.32	0.11	0.724
M2	175	0.35	0.04	0.724
M2	200	0.30	0.11	0.724
M3	25	0.70	—	0.724
M3	50	0.68	—	0.724
M3	75	0.85	—	0.724
M3	100	0.84	—	0.724
M3	125	0.94	—	0.723
M3	150	0.98	—	0.723
M3	175	1.00	—	0.720
M3	200	1.00	—	0.719
M4	—	—	—	0.724
